# Populations at Risk for Alveolar Echinococcosis, France

**DOI:** 10.3201/eid1905.120867

**Published:** 2013-05

**Authors:** Martine Piarroux, Renaud Piarroux, Jenny Knapp, Karine Bardonnet, Jérôme Dumortier, Jérôme Watelet, Alain Gerard, Jean Beytout, Armand Abergel, Solange Bresson-Hadni, Jean Gaudart, for the FrancEchino Surveillance Network

**Affiliations:** Aix-Marseille University, Marseille, France (M. Piarroux, R. Piarroux, J. Gaudart);; University College London, London, UK (J. Gaudart);; Franche-Comté University–University Hospital, Besançon, France (K. Bardonnet, J. Knapp, S. Bresson-Hadni);; University Hospital, Clermont-Ferrand, France (A, Abergel, J. Beytout);; Hospices Civils de Lyon, Lyon, France (J. Dumortier);; University Hospital Nancy, Nancy, France (A. Gerard, J. Watelet)

**Keywords:** Echinococcus multilocularis, epidemiology, communicable disease prevention and control, alveolar echinococcosis, rural health, zoonoses, agricultural workers diseases, risk factors, France, parasites

## Abstract

During 1982–2007, alveolar echinococcosis (AE) was diagnosed in 407 patients in France, a country previously known to register half of all European patients. To better define high-risk groups in France, we conducted a national registry-based study to identify areas where persons were at risk and spatial clusters of cases. We interviewed 180 AE patients about their way of life and compared responses to those of 517 controls. We found that almost all AE patients lived in 22 *départements* in eastern and central France (relative risk 78.63, 95% CI 52.84–117.02). Classification and regression tree analysis showed that the main risk factor was living in AE-endemic areas. There, most at-risk populations lived in rural settings (odds ratio [OR] 66.67, 95% CI 6.21–464.51 for farmers and OR 6.98, 95% CI 2.88–18.25 for other persons) or gardened in nonrural settings (OR 4.30, 95% CI 1.82–10.91). These findings can help sensitization campaigns focus on specific groups.

Alveolar echinococcosis (AE) is caused by the larval stage of the fox tapeworm *Echinococcus multilocularis*. In human infections, after a person ingests eggs, the metacestode cells of *E. multilocularis* proliferate in the liver, inducing a hepatic disorder mimicking liver cancer (*1*). Complete resection of liver lesions is possible in only one third of the cases, and parasitostatic and sometimes parasiticidal (*2*) treatment is available with benzimidazole compounds (albendazole or mebendazole). AE, observed only in the Northern Hemisphere, is linked to environmental features, such as land use for cattle breeding (pastures), which promotes high densities of rodents (main reservoir for the parasite) and thus a high prevalence of infection in foxes, which increases the environmental reservoir of the parasite (*3*,*4*). That the intermediate and final hosts of the parasite are members of wildlife species, largely explains why AE is an occupational disease of farmers and especially of cattle breeders (*4*). Individual risk factors vary greatly, however, depending on the country (*1*).

In Europe, the main AE-endemic areas are north of the Alps, primarily in Switzerland, France, Germany, and Austria, but recent studies showed that AE has spread during the past 20 years (*5*,*6*). Human AE cases have been diagnosed in countries previously considered free of the infection, such as Poland, Slovakia, Lithuania, Slovenia, Belgium, and Hungary (*7*,*8*). Molecular typing of *E. multilocularis* specimens collected in Europe showed that the European AE focus can be drawn as a core located in central Europe, flanked by neighboring regions where the parasite is less genetically diverse (*6*,*9*). In addition to the centrifugal spread of the disease, some epidemiologic studies also showed a significant trend of an increase in human AE incidence in some previously known foci, for example, in Switzerland (*10*). Schweiger et al. hypothesized that in Switzerland the increase in the fox population in rural and urban areas and high prevalence of *E. multilocularis* in foxes led to an increase in the infection risk for humans and the emergence of AE 10–15 years after infection increased in foxes (*10*).

France represents the western border of the European focus of AE and accounted for 235 (42%) of the 559 patients recorded in Europe during 1982–2000 (*7*). Hegglin et al. (*11*) have pointed out that AE is poorly known in France (only 88 [17.6%] of 500 interviewed persons were aware of it). This study reinforced the conclusion that better information is needed to identify at-risk populations. In particular, to avoid alarming the general population, we need to accurately define areas where persons are at risk for AE, identify exposed populations, and clarify behavior associated with AE contamination. Since the EurEchinoReg project (*7*), the FrancEchino Network has maintained a registry in France of AE cases, with the support of the French National Institute of Public Health Surveillance (Institut de Veille Sanitaire) (*12*). From 1982 through 2007, this registry helped identify 407 new patients in France (*13*). We present the results of a registry-based study in which we aimed to better define high-risk target groups for prevention campaigns.

## Materials and Methods

### FrancEchino Registry 

Initiated by the EurEchinoReg Network, the FrancEchino Registry actively gathers information about AE patients and related data observed in France since 1982 as described (*12*). During 1997–1998, French AE patients were found by sending questionnaires to all hospital departments of medicine, radiology, and abdominal surgery that look after patients with AE, to all pathologists and parasitologists in France, and to all public hospital pharmacies that could deliver albendazole or mebendazole for treatment. If no response was received to 2 mailed questionnaires, telephone calls were made. Clinicians, radiologists, pathologists, pharmacists, and biologists were asked to report all suspected AE patients diagnosed since 1982, that is, patients who had a positive specific serologic test result, a compatible imaging result, characteristic histopathologic features, and had received albendazole or mebendazole treatment for >30 days. During 1998–2007, the database was updated every 2 years following the same procedure. Previously unreported patients were also identified with the support of national medical societies (French societies of infectious diseases, parasitology, gastroenterology, digestive surgery, and the French association for the study of the liver). All suspected cases were further investigated by interviewing physicians using a questionnaire that addressed the patient’s AE clinical history.

Patients were classified into the following 4 groups, according to the recommendations of the World Health Organization Informal Working Group on Echinococcosis (*14*,*15*): “1) possible case—any patient with compatible clinical and epidemiologic history and imaging findings, *or* serology positive for AE; 2) probable case—any patient with clinical and epidemiologic history and imaging findings, *and* serology positive for AE with 2 tests; 3) confirmed case—the above, plus a) histopathology compatible with AE and/or b) detection of *E. multilocularis* nucleic acid sequences in tissue obtained through surgery or percutaneous biopsy; 4) all other patients were excluded from the study.”

### Questionnaires and Data Analysis

Patients with possible, probable, or confirmed AE were further investigated by a questionnaire addressing the epidemiologic context of their infection. Questions explored the patient’s life, using each past address to search for risk factors. Six binary questions were asked about behavior regarding picking wild berries, eating raw salads, hunting, having a kitchen garden, and having contacts with dogs and foxes. Interviews were carried by telephone by a physician or during a medical consultation. To assess behavior of the general French population, these questions were also included in a survey conducted by Ipsos Observer (Paris, France), an opinion poll marketing company (Certified ISO 9001: 2000 Bureau Véritas Certification), following the quota method (*16*). Quotas were calculated on the basis of sex, age (>15 years), occupation, and location, according to the French National Institute of Statistics and Economic Studies. Interviews were conducted on January 7, 2008. The sample size included 517 persons.

Each questionnaire contained data about age, sex, and socioprofessional characteristics and “commune” (smallest French administrative unit) of living. Nine factors were taken into account for analysis: the 6 above questions, occupation (previous occupation for retired persons), living in rural setting or not, and living in a *département* (second largest administrative unit in France after province) located in an area where persons were at risk for AE (DAR). A *département* where a cluster of cases occurred, identified with SaTScan software (Kulldorff, Boston, MA, USA; and Information Management Services, Inc., Rockville, MD, USA) (*17*), was considered a DAR. SaTScan moved an elliptic window of increasing diameters over the studied region (maximum size allowed for the smallest diameter was 120 km, whereas the largest diameter was not limited) and compared the observed AE case numbers in the window with the expected number under the null hypothesis, that is, the random location of cases among all *départements.* Statistical significance was obtained through Monte Carlo testing (results of the likelihood function were compared with 999 random replications of the dataset generated under the null hypothesis, following Kulldorff's approach) (*18*,*19*).

Data were recorded anonymously by using ACCESS 2000 (Microsoft Corp., Bellevue, WA, USA). Data obtained from the Ipsos Observer survey were weighted to reduce the bias due to the quota sampling. Incidence rates were referring to the mean between 1990 and 1999 national census. For univariate analyses, the Wilcoxon test and Fisher exact test were used, respectively, for quantitative and qualitative variable analysis.

Classification and regression tree (CART) multivariate analysis was used to identify behavioral groups related to AE risk. CART is a nonparametric and nonlinear regressive approach developed in the 1980s by L. Breiman (*20*,*21*). CART classified persons according to the outcome binary variable—AE patient/non–AE patient. Among all covariates, CART analyzed each possible threshold to split the sample in 2 opposite homogeneous groups. This process was recursively repeated until an optimal criterion was reached. 

When only the main covariates were kept, the process enabled a tree to be built in which the terminal classes were groups with common behavior. Because behavior covariates have been known to share collinearity (*22*), CART led to the building of behavioral groups that avoid this bias. To quantify the relationship between the behavioral classes and the disease, we estimated odd ratios (ORs) using a logistic regression. Statistical tests and CART analysis were performed by using R2.13.0 (R Foundation for Statistical Computing; http://cran.r-project.org/).

## Results

**Figure 1:**
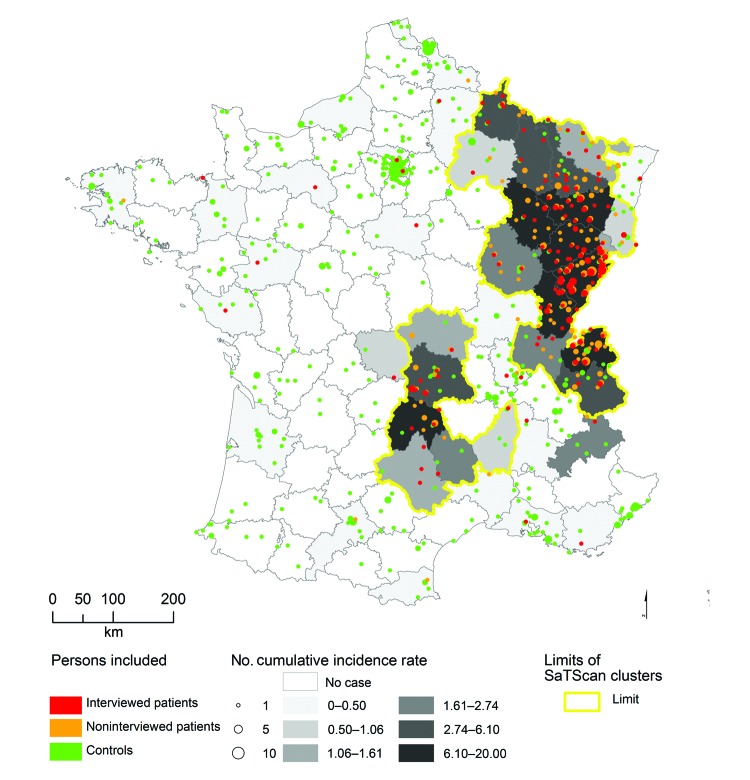
Location of patients, controls, and areas in France where persons are at risk for alveolar echinococcosis. The main area for human risk is located in eastern France and includes the *départements* (second largest administrative areas in France) where persons are at risk for alveolar echinococcosis of clusters 1, 2, and 4 as defined by SatScan analysis (Kulldorff, Boston, MA, USA; and Information Management Services, Inc., Rockville, MD, USA). Clusters 3 and 5 are located in the mountains of Massif Central and constitute the second area where persons are at risk.

*Département* of residence could be accurately determined for 399 of the 407 patients identified by the FrancEchino Network. SaTScan identified 5 significant high risk clusters (p<0.001for each cluster), including 22 *départements*. These 5 clusters have been gathered into 2 separate at-risk areas, an area located in eastern France and the other in the Massif Central, where 84% and 10% of the total of the French AE patients, respectively, are found (Figure 1). Taken altogether, they included 8,900,000 inhabitants, representing 15% of the French metropolitan mean population for the period. In these areas where persons were at risk, the risk of contracting AE was particularly high compared to the risk in the rest of metropolitan France (relative risk 78.63, 95% CI 52.84–117.02).

Because many patients who received a diagnosis of AE during the 1980s and the 1990s were lost to follow-up or had died, only part of the cohort could be interviewed. We obtained 180 questionnaires that could be analyzed, which showed 111 confirmed, 61 probable, and 8 possible cases. Interviewed patients were not significantly different (p>0.289) in terms of diagnosis status, age, sex, occupation, region of living, and type of *commune* from the whole set of patients, except that they were more likely to have been diagnosed after 1994 (p = 0.025).

Of these patients, 164 (91%) were still living in a DAR (Figure 1), 163 (91%) were living in the same *département* as they did 15 years before (144 [80%] in the same village) and 121 (67%) spent their entire life in the same *département*. Seven patients (4%) used to live in a DAR but had left it at diagnosis time for a median time of 11 years (95% interquantile central interval 0.6–26.8). All 7 used to come back into DARs every year to spend holidays. Altogether, the 171 patients who had lived in DARs stayed a median time of 56 years (95% interquantile central interval 12.7–81.5) in DARs before the AE diagnosis was made.

Of the 9 remaining patients who never lived in a DAR, 3 were living <50 km from the border of a DAR, 4 often traveled to DARs for summer holidays, and only 2 had little or no contact with DARs. The first person, who lived in Normandy (northwestern France), spent only 3 months in a DAR for military duty and had not been back for 34 years, and the second person, who lived in northern France, occasionally traveled to DARs, but only for skiing during winter vacations.

Once data were weighted, the IPSOS Observer–interviewed group (controls) accounted for 566 persons. It differed from the whole patient group by mean age (OR 44.9, 95% CI 43.36–46.35 vs. 58.09, 95% CI 56.78–59.40, p<0.001) and geographic location (only 16% living in a DAR vs. 94%, OR 77.92, 95% CI 48.32–131.04, p<0.001) but not for sex ratio (0.92 vs. 1.07; p = 0.27). Univariate analysis showed a significant difference (p<0.001) between interviewed patients and controls for all of the following factors: agricultural occupation (35% vs. 5%; OR 9.58, 95% CI 5.82–16.06), living in DAR (91% vs. 16%; OR 52.37, 95% CI 29.60–98.59) and in rural communes (communes with continuous dwellings with <2,000 inhabitants, 60% vs. 25%; OR 4.51, 95% CI 3.13–6.54), having a kitchen garden (92% vs. 63%; OR 6.98, 95% CI 3.91–13.39), eating raw salads (77% vs. 55%; OR 2.71,. 95% CI 1.83–4.08), having handled a fox at least once (29% vs. 13%; OR 2.69, 95% CI 1.76–4.09), or having a dog (81% vs. 62%; OR 2.59, 1.70–4.03). By contrast, the proportion of hunters did not differ significantly between persons with AE and controls (17% vs. 14%; OR 1.21, 95% CI 0.74–1.95, p = 0.4) and those in the AE group were equally likely to eat raw wild berries as controls (91% vs. 91%; OR 1.04, 95% CI 0.57–2.04, p = 1). The subgroup analysis that compared patients to controls inside DARs showed similar results (Table 1).

**Table 1:** Univariate analysis of studied behavior and area of living and risk for alveolar echinococcosis, France, 1982–2007*

Variable	Lived in DAR					Lived outside DARs			
	No. (%) patients	No. (%) controls	OR (95% CI)	p value		No. (%) patients	No. (%) controls	OR (95% CI)	p value
Total no.	164	92				16	474		
Had agricultural occupation	**62 (38)**	**7 (8)**	**7.33 (3.13–20.00)**	**<0.001**		1 (6)	23 (5)	1.31 (0.03–9.25)	0.558
Had kitchen garden	**152 (93)**	**64 (70)**	**5.50 (2.52–12.66)**	**<0.001**		**14 (88)**	**292 (62)**	**4.35 (0.98–39.90)**	**0.037**
Lived in rural/urban commune	**105 (64)**	**27 (29)**	**4.26 (2.39–7.75)**	**<0.001**		3 (19)	114 (24)	0.73 (0.13–2.72)	0.772
Had dog	**133 (81)**	**56 (61)**	**2.75 (1.49–5.09)**	**<0.001**		13 (81)	297 (63)	2.58 (0.70–14.30)	0.187
Handled fox	**50 (30)**	**12 (13)**	**2.91 (1.42–6.41)**	**0.002**		3 (19)	64 (14)	1.48 (0.26–5.58)	0.469
Ate raw wild salads	128 (78)	61 (66)	1.80 (0.98–3.31)	0.054		10 (63)	249 (53)	1.50 (0.49–5.12)	0.459
Went hunting	28 (17)	15 (16)	1.06 (0.51–2.27)	1		2 (13)	65 (14)	0.90 (0.10–4.06)	1
Ate raw wild berries	149 (91)	84 (91)	0.95 (0.33–2.50)	1		15 (94)	430 (91)	1.53 (0.23–66.05)	1

*DAR, *département* (second largest administrative area in France) located in at-risk areas; OR, crude odds ratio; commune, smallest administrative unit in France. **Boldface** indicates statistical significance.

**Figure 2:**
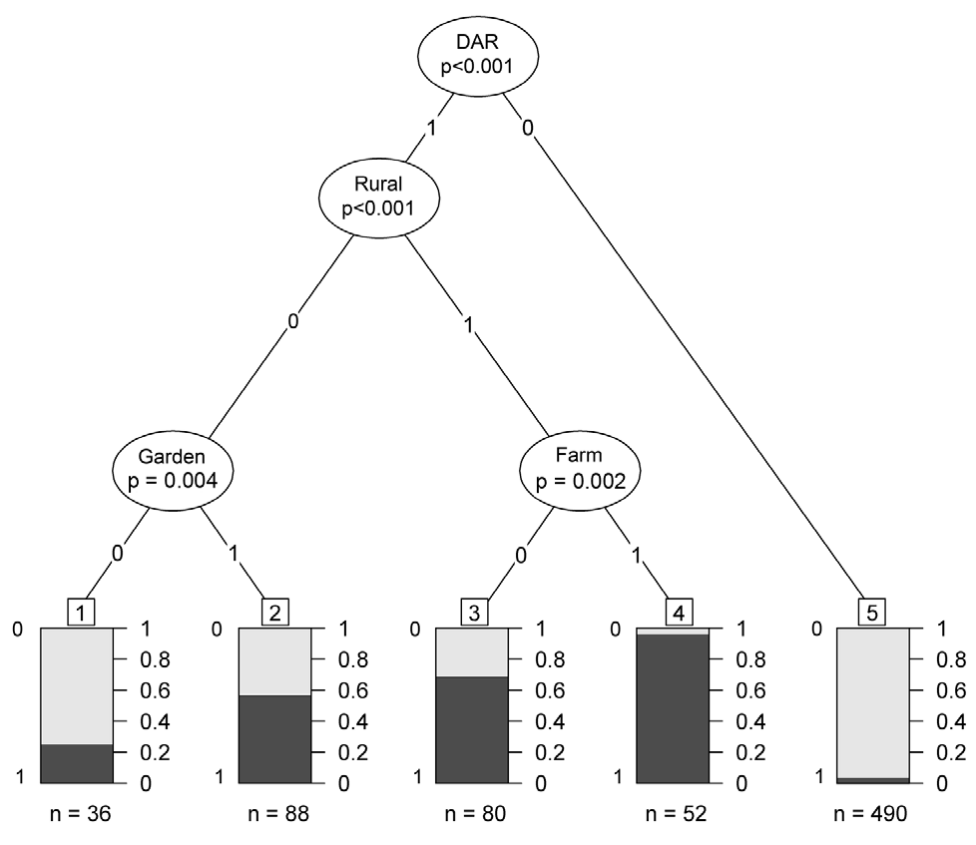
Significant results by multivariate analysis using classification and regression tree analysis to determine risk for alveolar echinoccosis in France, 1982–2007. Black indicates patients; gray indicates controls; class number is enclosed in a square. When the last step of analysis was not significant, terminal classes were aggregated at the upper level. Patients appeared predominant in 4 terminal classes: class 1 represented persons who live in an urban (or semiurban) environment in a *département* (second largest administrative area in France) where persons are at risk for alveolar echinococcosis (DAR) and do not have a kitchen garden, class 2, persons who live in similar areas but have a kitchen garden; class 3, nonfarmers who live in rural areas in a DAR; and class 4, farmers who live in the same environment; class 5, mostly persons who live in *départements* (second largest administrative areas in France) where humans are not at risk.

Multivariate analysis with CART (Figure 2) led to a definition of 5 classes of persons with a significant level of risk. The class with the lower risk corresponded to all persons living outside the 22 DARs previously defined, whatever their habits, occupation, and site where they used to live (rural or urban). Compared with our reference class (persons living in nonrural communes inside DARs and having no kitchen garden), they exhibited low risk (OR 0.097, 95% CI 0.039–0.250, p<0.001, Table 2).

**Table 2:** Analysis of behavioral classes and risk for alveolar echinococcosis, France, 1982–2007*

Class no.†	OR (95% CI)
1	1
2	4.30 (1.82–10.91)
3	6.98 (2.88–18.25)
4	66.67 (6.21–464.51)
5	0.097 (0.039–0.250)

*OR, odds ratio; DAR, *département* (second largest administrative area in France) where persons are at risk for alveolar echinococcosis. †When the last step was not significant, classes were aggregated at the upper level. Class 1 is the reference class: persons living in an urban (or semiurban) environment, in a DAR and having no kitchen garden; class 2, persons living the same environment in DAR but having a kitchen garden; class 3, nonfarmers living in rural settings, in DAR; class 4, farmers living in the same environment; class 5, persons living in *départements* where persons were not at risk.

Among persons who lived in a DAR, having a kitchen garden was associated with an increasing risk for persons who lived in nonrural communes (OR 4.30, 95% CI 1.82–10.91, p = 0.004). However, the risk was higher for persons living in rural communes (OR 6.98, 95% CI 2.88–18.25, p<0.001), and among them, even higher for those with agricultural occupations (OR 66.67, 95% CI 6.21–464.51, p = 0.002).

## Discussion and Conclusions

Although it has long been known that most of eastern France and, at a lesser degree, the Massif Central are major foci of AE in Europe (*7*), few data have been available concerning the specific risk factors for this disease in the French population. Our study confirmed that almost all patients with AE live in rural areas in eastern and central France. In addition, a heterogeneous geographic distribution of AE has been reported in numerous foci, such as in Hokkaido, Japan, where humans cases spread from the various areas where infected foxes were introduced in the 1920s (*23*); in the People’s Republic of China, where the disease is endemic to only the western and northern provinces and the autonomous regions (*24*); and in other European countries such as Poland, Germany, Austria, and Switzerland (*7*). 

In our study, mapping the locations of almost all patients in France who received a diagnosis of AE over 26 years and detecting clusters of patients allowed us to accurately identify the current areas in France where human AE is endemic. Less than one tenth (17/180) of the patients who were interviewed did not live in the same *département* at diagnosis and 15 years before, and only 5% (9/180) had never lived in the DARs defined by our spatial analysis. Taking into account the rarity of the disease in France (15 new patients/year until 2007) (*12*,*13*), this means that <1 patient per year received a diagnosis of AE among 48 million French citizens residing outside DARs during the study period (mean French metropolitan population was 57,500,000 for the study period, with only 8.9 million living in DARs). This low incidence of AE in most regions of France may have been exaggerated by misdiagnosis in areas where the disease is poorly known by physicians. However, the chronicity and the severity of the disease associated with the performances of diagnostic imaging and serologic testing, which greatly improved during the last decades, make a persistent misdiagnosis of AE lesions unlikely (*25*).

Because regulations in France require that albendazole be dispensed only in public hospital pharmacies, we can be reasonably confident that our detection of cases, using a multidisciplinary approach, was complete. Therefore, the clustering of almost all French cases in a few specific locations demonstrates the importance of the place where persons live and had lived in the risk assessment of AE and the precautions that have to be taken when generalizing incidence numbers to the total population. Human data reflect the parasitic transmission a few years before diagnosis; they are not sufficient to determine the current situation regarding the risk of transmission and must be combined with frequently updated animal data.

In contrast with the highly clustered location of human patients, the main hosts involved in the life cycle of AE (foxes, dogs, voles) are found almost everywhere in France (*26*,*27*). Until the end of the 20th century, infected foxes had never been observed—but also not systematically searched for—outside the areas to which AE is endemic in humans, that is, eastern France from the northeastern border to the southern Alps with a limited focus in Massif Central (the Cantal area) (*26*). Our findings fit well with such a distribution of the infected foxes, although they show a substantial extension of the Massif Central focus toward the southeast and northeast. However, a recent screening campaign of the fox population showed the existence of infected foxes in northwestern France, including in Paris and its suburbs, and reaching the English Channel coast (Normandy region) (*28*). In our series, 2 patients from northern and northwestern France were found to be infected although they had almost never traveled to DARs. Even though AE prevalence in foxes is usually lower outside DARs than in them (leading to a lower risk for humans), these 2 patients could be an early indication of a broader extension of AE in the French population.

Notably, AE lesions develop slowly in humans, leading to a long period of latency between the initial infection and the diagnosis. Because of this latency period, AE in humans would increase only 10–15 years after *E. multilocularis* infection incidence increased in foxes. Such a phenomenon has already been observed in Switzerland (*10*), and recently Takumi et al. hypothesized that this could happen in the Netherlands before 2020 (*29*). Moreover, foxes are now often living in urban areas (*30*–*32*) and have a high incidence rate of *E. multilocularis* infection in some cities located in AE-endemic areas (*30*), so urban gardeners could have a higher risk in the future. Prevention campaigns must target this group in addition to rural inhabitants in regions where AE is endemic in humans and foxes.

Our findings identified many factors associated with patients’ way of life such as having an agricultural occupation or having a kitchen garden, 2 factors that increase contact with soil possibly contaminated by *E. multilocularis* eggs. Having a dog and/or handling foxes, 2 risk factors associated with the spread of *E. multilocularis* eggs by their main definitive hosts, were also significant variables. In Germany, a case-control study also found that farmers were at highest risk (2*2*), whereas gardeners were found to have additional risk only if they grew leafy or root vegetables.

The most commonly alleged source of infection emphasized in public media, that is, “eating wild berries,” was not found to be an additional risk in our study. Conversely, we found that almost everyone did pick berries (91% in both the control and AE groups). If such behavior would have been a noticeable risk factor, some cases should have been diagnosed every year among the millions of the French city dwellers who go to DARs for hiking and tourism. In Germany, Kern et al. (*22*) could not rule out the category “eating unwashed strawberries” when building an individual risk score for AE. However, this factor was the least significant included in the score they developed. Similarly, our findings, as well as those of Kern et al., did not link AE with hunting. In contrast, a previous study in Austria (*33*) identified hunting as the most notable observed risk factor (OR 7.83, 95% CI 1.16–52.77). This Austrian study included only 21 cases, preventing he authors from analyzing confounding factors such as living in a rural area. This study did not find that owning a dog led to a statistical risk.

Dog ownership has been noted in many countries (Saint-Laurent Island, Alaska, USA [*34*], China [*35*–*37*], and Turkey [*38*]). In Germany (*22*), this was a major risk only when persons owned dogs that “roamed outside,” “killed game,” or “were irregularly dewormed.” Overall, CART analysis was of particular interest in our process to determine AE risk factors. It is obvious that a person living in a rural area in France is more likely to own a dog, have a kitchen garden, and work in agriculture than a person living in a city. Although the method only indicates broad trends, CART could bypass the problem of collinearity between variables and allow the main profiles of persons to be defined on the basis of specific level of risk of infection.

In conclusion, determining who is at risk of acquiring AE will enable prevention campaigns to be focused on specific population groups. Tools and recommendations are already available to limit the risk of infection in humans (*23*). These tools include frequent hand washing, proper food handling, pet deworming, discontinuing vegetable gardens, and avoiding contact with foxes. Using bait to deworm foxes has also been proposed (*30*). Because AE remains mostly clustered in geographically distinct areas (and persons who spend only vacation time in these AE-endemic areas do not appear at high risk), sensitization campaigns should be aimed at persons who live in AE-endemic areas, especially those who have an agricultural occupation, have a garden, or live in rural settings. Local outlets, such as rural medical offices, pharmacies, and communal administrative information bulletins as well as local newspapers and radio and TV channels could convey awareness messages. Placing general advertisements in national media might be counterproductive by alarming persons not at risk for the disease and by focusing on the most sensational or supersensitive information (such as the potential danger of wild berries) (*39*,*40*), while overlooking more obvious measures to avoid contamination, such as pet deworming and hand washing after gardening or playing with pets. Nevertheless, the situation might change, and screening for human AE cases and continuous monitoring of fox infections could enable this strategy to be adapted to new foci as necessary**.**
